# From trauma to incarceration: exploring the trajectory in a qualitative study in male prison inmates from north Queensland, Australia

**DOI:** 10.1186/s40352-016-0034-x

**Published:** 2016-04-01

**Authors:** Bronwyn Honorato, Nerina Caltabiano, Alan R. Clough

**Affiliations:** 1 James Cook University, Room E1.003A, McGregor Road, Smithfield, Cairns, 4870 QLD Australia; 2 Discipline of Psychology, College of Healthcare Science, James Cook University, Cairns, QLD Australia; 3 Australian Institute of Tropical Health and Medicine, James Cook University, Cairns, QLD Australia

**Keywords:** Male prison inmates, Incarceration, Trauma, Violent offending

## Abstract

**Background:**

There were approximately 34,000 prisoners incarcerated in Australian correctional centres as of 2014. The most common offence type for these prisoners was ‘acts intended to cause injury’, comprising 18 % of the total offences. Of the various risk factors for violent offending and incarceration identified in international research, trauma - either single events or ongoing; and substance abuse - which is commonly associated with violent behaviour across many cultures, are major contributors.

**Method:**

This paper analyses qualitative data from 11 in-depth interviews with inmates from a high security male correctional centre in QLD, Australia. The aim of the study was to explore risk factors for violence and incarceration for men from far north Queensland.

**Results:**

A common trajectory to violent offending and incarceration was identified for these prisoners, including: childhood/adolescent trauma; a lack of support or treatment for trauma experiences; substance abuse to mask the pain; and a ‘brain snap’ precipitating a violent offence.

**Conclusion:**

Further research is required into factors leading to violent offending and incarceration generally. In particular early detection and intervention for trauma victims is imperative in order to reduce exposure to such a harmful trajectory from trauma to incarceration.

## Background

On average, just over 34,000 inmates were incarcerated in Australian correctional centers in 2014, with the majority (91 %) being male (Australian Bureau of Statistics 2014). Indigenous Australians accounted for 28 % of the nation’s prison population as of June 2015, despite making up only 2.5 % of the general population (Australian Bureau of Statistics 2015). Violent offences, or specifically ‘acts intended to cause injury’ were the most common type of offence in 2013–14, accounting for 18 % of the total offences for all prisoners in Australia (Australian Bureau of Statistics 2014b).

Many individual, social and environmental factors influence the likelihood of an individual engaging in violent behavior and becoming incarcerated (Hawkins et al. 1992; Homel et al. 1999; Kelly et al. 2009). Risk factors are not static; rather they interact over an individual’s life course; and may be influenced by differences in culture, gender and other circumstances. One such risk factor for violent behavior and incarceration for many young men, is trauma (Carlson and Shafer 2010; Walsh 2007). Trauma may include physical illness or injury; harm; disability; torture; incarceration or persecution; relationship dissolution; job loss; migration/relocation; violence; and sexual abuse (Walsh 2007).

There is evidence that childhood trauma is a determinant of aggression in incarcerated populations. For example, in an Italian study of 540 prisoners, Sarchiapone et al. (2009) suggested that childhood trauma represents a developmental determinant that may interact with genetic factors to predispose prisoners to aggression. Further study is required, however, to generalize these findings to the wider, non-forensic, mixed-gender population. Additionally, Carlson and Shafer (2010) studied the trauma histories and stressful life events of 2279 inmates in Arizona, United States of America (USA). They found high rates of exposure to traumatic events, especially child abuse, across gender and ethnic groups. Other research shows youth involved in the criminal justice system typically have extremely high rates of trauma exposure from early life (Dierkhising et al. 2013; Ko et al. 2008). Furthermore, incarceration itself holds the risk of continued trauma and abuse, with traumatized youth more likely to reoffend as a juvenile or an adult, and to have poor long-term economic, academic and mental health outcomes (Justice Policy Institute 2009; Widom and Maxfield 1996).

In Australia, trauma experiences and Post Traumatic Stress Disorder (PTSD) are far more common among prisoners than among the general population (Australian Institute of Health and Welfare 2013). While there is limited information about the rates of trauma and PTSD for incarcerated Indigenous Australians, trauma has been found to play a pervasive role in their mental health burden (Atkinson et al. 2014).

Along with trauma, substance abuse is a major risk factor for aggression, violent behavior, and incarceration. The relationship between substance abuse and violence however, is exceptionally complex and moderated by a host of factors in the individual and the environment. Goldstein’s (1985) tripartite model suggests that substance use and violence are related in three ways: firstly, psycho-pharmacologically, whereby drug use effects crime due to the disinhibiting and disorienting effects of the drug on the body and mind; secondly, economically, as crime becomes a compulsion, a way to pay for drugs to abuse; and thirdly, a systemic contribution to crime whereby criminal behaviour becomes a normalised way of doing drug-related business (Goldstein 1985). Contemporary research recommends this tripartite model be refined to take into account the wide range of factors that influence the connection between drug use and crime, including the cultural context in which it operates (Bennett and Holloway 2009).

Findings from laboratory and empirical studies also support a strong temporal association between substance abuse and violence, with the use of alcohol and drugs consistently occurring prior to or during the commission of many violent acts (Boles and Miotto 2003). Furthermore, there is strong evidence for a subset of substance users who exhibit violent behavior to have a predisposition to aggressiveness starting in childhood (Boles and Miotto 2003). Substance abuse has been shown to be a risk factor for both violent behavior and incarceration for Australian men in various studies. For example Putt et al. (2005) found alcohol intoxication to be directly associated with violent offending for Indigenous men. Further, Hemphill et al. (2009) examined longitudinal data for predictors of youth violence, and found binge drinking to be a major risk factor for violent behavior 12 months later. Illicit drug use has also been found to be a risk factor for violent offending (Torok et al. 2012) and incarceration (Kenny and Lennings 2007; McKetin et al. 2014) for Australian men in various studies.

The current study reports on findings from 11 in-depth, qualitative interviews conducted with male inmates at a high security prison in QLD, Australia. The overall aims of the study were to: gain a better understanding of the risk factors underlying violent offending and incarceration for this group of Australian men; and whether differences existed in these factors for Indigenous compared to non-Indigenous men.

## Methods

The research project had approval from the James Cook University (JCU) Human Research Ethics Committee (ref H5273) and Queensland Corrective Services (QCS) Research Committee. All participants provided their written consent to participate. QCS provided consent to publish the results of this research project.

### Participant recruitment

Research in the custodial setting must comply with all requirements, protocols and schedules of QCS. Accordingly, the correctional centre’s residential accommodation manager was delegated by QCS to conduct the recruitment of suitable participants, in line with the study participant selection criteria. The principal researcher (author BH) advised the residential accommodation manager that potential participants were required to be: i) physically well and mentally competent to participate; ii) able to understand and speak plain English; iii) sentenced or on remand for violent offences; and iv) aged 18 years and over. Each participant was given an information sheet and a written or verbal description of the project, and was asked to sign a consent form if they agreed to participate. The residential accommodation manager then contacted the principal researcher to arrange a suitable time to conduct one-on-one interviews. The principal researcher, together with an experienced research assistant, attended the centre and conducted interviews in a private room in the residential accommodation division. Prison officers were on hand at all times to ensure the safety of the researchers and the wellbeing of participants.

To minimise any distress arising from the interviews, and in accordance with QCS research committee and JCU ethics requirements, participants were advised that counselling was available following the interviews if required. Furthermore, participants were advised that, while interviews were strictly confidential, ethical standards and QCS research committee regulations regarding maintenance of confidentiality stipulate that if participants divulged information relating to plans for self-harm; harm to others; or previously undisclosed crime; then these details must be reported to prison staff.

### Data collection and analysis

Interviews were conducted using an unstructured ‘research yarning’ approach of listening, talking and observing, congruent with cultural processes for Indigenous and non-Indigenous participants (Bessarab and Ng’andu 2010). Participants were initially asked their age, marital status, education level and occupation prior to prison. Using a checklist to guide the interviews, participants were then asked to: “Tell us your story - how did you end up in prison?” This enabled the disclosure of rich accounts of their experiences in a non-threatening way. It also allowed the researcher flexibility to probe interesting and significant areas that emerged as the interview progressed. Various topics were explored, including participants’ childhood and family life; histories of violence and incarceration; attitudes towards incarceration; reasons for their offence/s; and thoughts on what may have kept them out of prison if circumstances were different. Where consent was provided by the participant, interviews were audio recorded and transcribed verbatim by the principal researcher (BH) or research assistant at a later date. Some interviews were not recorded, i.e. when participants preferred this or when circumstances did not permit. These interviews were transcribed from hand written notes. Interview data was then organized and coded into themes using NVivo qualitative data management software.

The coded data was then interpreted and analysed according to Interpretative Phenomenological Analysis (IPA), in order to try to understand the participants’ lived experiences and how they themselves made sense of their experience (Smith 2004). Once the transcripts were fully coded, two experienced researchers (co-authors AC and NC) reviewed and checked several samples of coding for consistency.

Interview data from a total of 11 participants was used for the current study. A relatively small sample size is acceptable for this type of challenging work due to the use of (IPA) methodology; with a focus on intensity and richness of information (quality) rather than quantity (Smith 2004). IPA began with close, line by line analysis (coding) of individual interview transcripts for each participant. Emergent patterns (themes) were identified, recognizing convergence, divergence, commonality and nuance for single, then across multiple cases. From the coded data, the researcher interpreted what it might mean for participants to have these concerns in the particular context. A structure was then developed, illustrating the relationship between the themes. This procedure was in accordance with the analytic process described by Smith (2004).

## Results

### Participant demographics

Demographics including age range, culture, marital status and employment are shown in Table 1. The participants’ education levels ranged from Grade 6 to Year 12, with the majority having completed either Year 9 or Year 10 (*n* = 7). Offences for which the participants were under remand or sentence at the time of interviews included: assault; grievous bodily harm; attempted murder, murder; intent to rape; rape; domestic violence and domestic violence breaches; armed robbery; robbery on the run; break and enter; and drug offences.

**Table 1:** Participant demographics

Age range					
19 – 44 years	(mean 33.3 years)				
Culture					
Indigenous	5	Non-Indigenous	6		
Marital Status					
Single	7	Partner	4		
Employment					
Fulltime	4	Casual	3	Unemployed/other	4

Four prominent recurring themes were identified within the interview data and organized into a simple four step trajectory as follows: 1. Trauma: including actual trauma and the impacts of trauma as a child or young adult; 2. A lack of coping skills or support to deal with the traumatic event or long term trauma; 3. Substance abuse to cover/mask the effects of the trauma; and 4. An act of spontaneous violence (described as ‘*brain snap*’, and ‘*losing it*’). These four overarching themes will each be explained in further detail including the participants’ own description of how they lived through and interpreted these experiences, and the possible meaning behind these statements.

### Trauma and impacts of trauma

#### Trauma

All of the participants interviewed reported experiencing trauma in their childhood or adolescence, ranging from one-off events to prolonged exposure. The types of trauma disclosed included: sexual abuse by family or others; family members being assaulted/sexually assaulted, kin committing suicide or being killed; and being subject to severe bullying.

The following quote comes from a participant who was subjected to violence and sexual abuse as a child, continuing well into his adolescent years. The abuse was then perpetrated by non-relatives within custodial settings. The trauma of his innocence and safety being compromised from such an early age began his trajectory into a prison life: “*… he was a bad alcoholic my stepfather, um that led to some pretty violent abuse and sexual abuse… this probably went on till I was in grade 8, when I was 13… When I’d gone from 17 to 18 [years old], you go from boy’s yard to mainstream, and then I was sexually assaulted pretty badly at [a previous correctional facility] when I was 18…” (Non-Indigenous, age 32).*

The lack of options for keeping a young person safe in custody are also highlighted by the following two participant’s statements. Ironically, while authorities were trying to provide protection, the young men’s safety ended up being compromised. Firstly, one participant recounts his ill-fated placement amongst a group of paedophiles which led to sexual assaults: “…*at one stage because I was so young they put me in the paedophiles yard, cos they said they couldn’t put me in with the mainstream population so they put me in a protected yard with all the paedophiles. That caused a couple of little things there [sexual assault in a previous correctional facility]” (non-Indigenous, age 42).* The second example also shows how a placement became a traumatic situation for the participant, from which he could not escape, however this time he was being abused by older boys in custody: *“And they put me in a dorm with older boys there who then abused me…And I couldn’t do anything about it, I couldn’t…” (Non-Indigenous, age 39).*

The experience of the following young man highlights the impact of long term trauma, living with the impact of being bullied by school mates and family members who should have protected him. Marginalization, a lack of acceptance from others, alienation from society and having no place of safety were precursors to his incarceration: “*I had a terrible time at school, and all through my younger years I was always picked on, even me brothers used to pick on me…but real, like I mean really badly bullying all the time… [I was] always in fights, so it was never, it wasn’t a good time for me whatsoever, growing up. And um [I] always found it really hard to gain acceptance off people… And like just to have one friend when I was growing up would have been good, but I didn’t have any …”(Indigenous, age 30).*

The next example demonstrates the traumatic situation of his father’s suicide and the participant’s inability to appropriately deal with this. He directly relates his current and past offending to his father committing suicide, which was a turning point in his life. Perhaps if his father was alive he would not have offended and his life may have had a different outcome: “*Yeah, since to be honest, since my dad, suicided himself, shot himself, then I started [offending]…My dad shot himself, in 1985, yeah.”(Indigenous, age 44)*.

At a very young age the following participant witnessed his father involved in a violent incident, an event for which the participant never received counseling. The time of year appears to be a trigger for his later offences, of which one serious violent incident occurred on Boxing Day [not detailed here]: “*I remember, I reckon I don’t know if it’s got any bearing on my offence, but I remember my fifth Christmas, when I was only five my dad did have that agro streak, everything like that, I did witness getting the living shit beat out of him by five other people who’ve come in to try and calm him down, but he went off…watching my dad get the living shit kicked out of him next to the Christmas tree, so…”(Non-Indigenous, age 23).*

#### Impacts of trauma

Untreated mental health issues, apparently resulting from trauma, were common to the study participants. The following excerpts emphasise issues of anger and psychosocial distress experienced, either at the time of the trauma or in the following months or years. These comments highlight that while participants may have had considerable insight into their problems and what had brought them about they did not have access, or the knowledge or services available, to enable them to receive adequate treatment for their psychological issues. For example, the following participant acknowledge that he had anger problems but did not know or did not have access to help for this: “*I had a problem but I never really talked to anyone. And I carried it around for years, I had like an anger problem and I don’t know…Yeah, just sort of seemed to carry a lot of anger around, when I should like, got help, to get me through it…And yeah, it was really hard…” (Non-Indigenous, age 43).* Untreated anger issues escalated to severe violent behavior for others, for example: *“Like I’ve always been suspended and expelled from schools for being violent… cos back when I was young…like, a trigger I could go from zero to 100 in a second, I got this really…sort of like the anger where you’d cry and you’d be shaking and that angry that you just want to destroy things…So there was times there, like I’ve choked other schoolmates in class, you know, assaulted the principal, assaulted teachers stuff like that.” (Indigenous, age 30).* Had this young man have received help, his trajectory in life may have been quite different.

The next two statements highlight again that the participants recognized they had trauma and anger issues, but did not receive counselling to ameliorate the effects of these events. The first participant alludes to the possibility of having PTSD, and seems to place blame on his parents and brother for the resulting problems with drugs, violence and jail: *“Yeah, post traumatic. Yeah. I was pretty angry with my parents too, at the time, for not having insurance…And my brother for burning it [the house] down.” (Non-Indigenous, age 39).* The family home burning down was the event that was perceived by the participant to have led to his offending and ultimate incarceration. Before that, he states that he was a ‘normal kid’, and that trauma and the impact of the feeling of being abandoned by his parents was instrumental in changing his life course: “*Um, oh we’ve had a fair share of dramas in my family, like we had a house burn down when I was a kid, and um so I, my parents weren’t around much, because they had to work because they didn’t have insurance…Um, yeah I was pretty much a normal kid, well I thought I was, wasn’t really…wasn’t really [delinquent]…no, not at that stage. But I think yeah, that house burning down put a lot of pressure on our family.” (Non-Indigenous, age 39).*

Had help have been available for the next young man, his anger issues and the unresolved trauma of his brother’s death, he may have avoided the addictive behaviors responsible for his later offending: *“And I started using, cos one of my brothers was killed, back when I was 15 and I had a lot of anger issues and I started yeah, like using drugs and drinking…*”*(Non-Indigenous, age 43).*

### Lack of support and coping skills

The following three statements highlight the lack of support and guidance available to these young men following the trauma they had suffered. They acknowledge that if they had have received some help, they may have not engaged in the destructive behaviors that ultimately led them to incarceration. For this first participant the lack of a father figure to support him was seen as a reason for his poor coping skills and eventual offending: “*And cos I didn’t have a fatherly figure around me, so well, I didn’t notice until now, like until now, well it hit me in jail, like I didn’t have a fatherly figure around for me to like guide me and stuff. So back then I wanted to try things myself, instead of like well if I reckon if I had a fatherly figure back then I wouldn’t be in here”(Indigenous, age 19).*

Here the next participant adhered to the social norm of men needing to be stoic and keeping their emotions in check, rather than seek help for psychological distress. Additionally, through his lack of cooperation, teachers found it difficult to control his behavior and he was basically left to do as he wanted: *“Yeah, you don’t cry or anything, you know, you just get along with it. But it really upset him as well [Dad], cos he never really talked to anyone as well, you know. He went through a real hard time as well…Yeah, but I sort of got that way that teachers didn’t even try and, I just did what I wanted to do, you know. They had no control over me, pretty much. I wouldn’t listen to anyone…”(Non-Indigenous, age 43).* This participant also felt that because he was so stubborn and didn’t listen, no one bothered to help him, again highlighting the feelings of abandonment that he had previously felt from his parents in his previous statement regarding his family home burning down.

A long history of chronic bullying and a lack of support and friendship, coupled with a desperation for acceptance and belonging led to the following participant’s willingness to overlook his friends’ engagement in criminal activities. Unfortunately this ensured that the he became embroiled in such activities leading to his ultimate incarceration: “*And that’s where I initially met some members of a motor cycle gang. And ah, I met them there and played football with them and they invited me to one of their parties…And you know, I felt like I, that thing that was missing out of my life, acceptance, and friends you know, stuff like that, I found that there. You know, and you know it’s everything I ever wanted. Just to be accepted for who I was, you know…Um, which I never had when I was a kid. So basically yeah I just found acceptance, and you know everything spiraled.” (Indigenous, age 30).* It didn’t matter that he had found friends who were involved in crime, he was just happy to be finally accepted by someone.

### Substance abuse

#### Substance abuse to mask the pain

Unresolved trauma led to anger issues and substance abuse for the study participants, as exemplified in the following statements. One participant explained his embarrassment at having other boys of similar age abuse him, his realization that nowhere was safe, that he had internalized his trauma without support or treatment, and that he used drugs for relief: *“Yeah, and I think the abuse and that, that I got there [boys home], I think that’s why I first started smoking dope, yeah…and I didn’t want to tell anyone [about the abuse] because it was so embarrassing.” (Non-Indigenous, age 39).* Here alcohol, rather than getting psychological help, became the way this participant coped with his grief: “*Oh, I guess just pressures of outside, there wasn’t really much pressure but it was more of a, my mum passed away and grief got the best of me. Instead of turning to help I just turned to alcohol, you know.” (Indigenous, age 39).*

Another participant found himself within a network of older men who were using drugs, highlighting the influence of peers/older men and the introduction to drugs. This soon became his way of coping with life’s stressors: “*I’d run away from home…I just found myself living with older blokes that were using and… I was pretty much flying, I started using needles and shooting up heroin. And for a long time it’s just, it just suppressed everything, like heroin’s just the sort of drug that pulls a warm blanket over you” (Non-Indigenous, age 32).* Similarly, the problems this participant was dealing with, including the gruesome circumstances of his sister’s murder, were dealt with by using heroin, first offered to the participant by his peers: “*… I first had my first lot of heroin in 1994, my older sister was raped and murdered at the time, and someone offered me some heroin when I was in jail, and that’s what started it…not just that, a lot of other things happened around the same time, mum passed away shortly after.” (Non-Indigenous, age 43).*

The following example also demonstrates the young adult’s reliance on peers for advice rather than counseling, and the use of drugs to cope with his problems: “*And like I was panicking and shaking [running away from police] so um, I learned how to smoke…what’s that…marijuana? Yeah, I heard some of them boys said “Oh it’s cool yah everything down”, so then I smoked that…I didn’t want to try that, but when they were talking to me about marijuana, I was like “oh, yeah”. So I tried marijuana, I loved it cos it just make my body relax, settle my blood pressure, and all them one. Make me think of, not think of the negative stuff, just zone out like just like see some like funny stuff, like yeah…” (Indigenous, age 19).*

Drug use was acceptable in the profession that the next inmate chose, so it really did assist to cover up his issues without him standing out from his workmates or peers. For a while it helped him to self-medicate from his trauma: “*Yeah well pretty much like, my lifestyle…I started using drugs and that when I was 15, 16 years old. And then I became a chef…my lifestyle sort of went hand in hand. I used to use a lot of drugs and that, just to sort of, I don’t know, just to mask everything.” (Non-Indigenous, age 43).*

While initially drugs appeared to alleviate the following participant’s problems, he found that when not using, his problems stood in stark contrast to how he imagined them when using: *“I tried drugs and sort of those problems just went away. I dunno if I took it intentionally to, but they just disappeared slowly, and they come back twice as hard when I got off em.” (Non-Indigenous, age 42).*

#### Substance use as a reason for offending

The following participants recognized that alcohol and drug use was a major cause of their violent behavior and ultimate incarceration, however the first two reasoned that it was not how they would normally behave: *“From that same shit now, sorry for my language, but from the same stuff now…I try things like I don’t normally do, like [sexual assault], violence and stuff, stealing…Yeah. I was drunk…when I was on the run I started trying drugs.” (Indigenous, age 19).* The second participant also blamed alcohol, however was very strong in his opinion that it was not an acceptable excuse. He is aware that he had his own free will but chose to drink and offend: “*Alcohol’s a massive one too, underline alcohol. Alcohol’s the worst mate…you do stupid shit on alcohol mate, you know. It’s the worst drug out there…I mean you could go further and you could say a lot of times alcohol is the biggest factor; it was a factor with me on this particular instance. I was drunk and there was things going on in my life, but the thing is, so it’s no excuse, coming down to alcohol.”(Non-Indigenous, age 30).*

In this third example, the participant found himself spiraling from alcohol use into heavy drug use and offending, with the realization that the substance use was not in fact helping the situation: “*Everything was good for the first year or two but eventually everything spiraled out of control and I started drugs and doing it a lot…Started doing violent things…I only started drinking when I was 17, 16…and smoking marijuana and stuff like that at that age, eventually that turned into ecstasy and cocaine and speed…Yeah, that’s when I started getting into dealing drugs, and …… and you know, anything and everything I could possibly do.” (Indigenous, age 30).*

While substance abuse was consistently mentioned by all participants as a contributing factor, or even the perceived cause of their violent offending, violent behaviour was often unplanned when the individual was under the influence of alcohol or drugs. Having a *‘brain snap’*, *‘losing it’*, or ‘*exploding*’ are some of the ways inmates described the moment of committing their violent offence or offences that led them to incarceration.

### ‘Brain snap’/‘losing it’

The following extracts are typical of those reporting this experience or uncontrolled acts of violence: “*And then one day it was like 15 years after it happened, and one day I just sort of lost it, and yeah killed someone … I don’t know, it was just like something snapped, I had no control over it…and when I realised what had happened I couldn’t believe it, like… just something built up, built up and built up and just whoosh, come straight out…I’ve always had a problem with that…I bottle a lot of stuff up and I’m still trying to find ways of letting it out without taking it out on anyone else in front of me, you know”(Non-Indigenous, age 43).* Had this participant had counselling and developed ways of dealing with his anger issues, the outcome could have been quite different. While he felt the offence was outside of his control, it served to ease some of his psychological distress, by lifting a weight off his shoulders.

Not unlike the previous example, there is a distinct lack of control and inappropriate way of dealing with the anger issue from the following three participants: “*… it’s ridiculous yeah, when I was confronted by him, who knows, I wasn’t thinking, like your brain is reduced to thinking in simplest terms mate…I’m a happy drunk…Not on that night but…a trigger point for me is when someone…threatens me. Gets in my space, do you know what I mean?” (Non-Indigenous, age 30).* Combined with a lack of knowledge as to where help could be obtained, participants are often diverted to inappropriate ways of coping as the following two passages indicate: *“Ah, I tried to kill my family…what happened was I kind of bottled it up instead of talking to people, cos I didn’t really know there was help outside…I bottle stuff up until I get drunk and then I just explode.”(Indigenous, age 30)*; and *“I was drunk, I was under the influence of alcohol and drugs, and I just exploded aye” (Indigenous, age 26)* Again, the behavior caused by the use of alcohol in combination with anger management issues, tends to be a dangerous mix for some people.

It is noteworthy that there were no substantial differences apparent between Indigenous and non-Indigenous participants with regard to these common themes and the trajectory to violent offending and incarceration. While there may be particular subtle differences that could be revealed with further interviewing, the course for all inmates who participated in this study was very similar.

## Discussion

It is evident that for many of the participants in this study there is a common trajectory from childhood or adolescent/early adulthood trauma to incarceration. This trajectory consistently included the following elements: trauma, including impacts of trauma; a lack of support; substance abuse to mask the pain, and as a reason for violence; and a brain snap, or uncontrollable anger and violence; resulting in incarceration. The findings for this trajectory also concurs with an early literature review from the United States of America, showing an association between PTSD, violence, trauma and substance abuse (Dunnegan 1997).

In the current study, a traumatic event or ongoing trauma as a young person was commonly identified as a catalyst for future violence. The types of trauma commonly described by the inmates included: serious ongoing bullying; death of a close family member; witnessing violence towards others; and other one-off-events such as the family home burning down. The impact of such trauma for these men from north QLD is consistent with findings from literature available elsewhere. For example, a 2013 study conducted in the USA found that up to 90 % of justice-involved youth had exposure to some type of trauma, typically beginning in early life, in multiple contexts, and persisting over time (Dierkhising et al. 2013). Supporting this, youth in detention and incarceration often have histories of complex trauma, such as poly-victimisation, life-threatening accidents and interpersonal loss (Ford et al. 2012).

According to Walsh (2007), the outcomes of trauma depend greatly on whether victims are provided with support, including comfort, reassurance and safety from others. Strong connections, with trust that others will be there for them when needed, counteract feelings of insecurity, helplessness and meaninglessness. Trauma survivors blocked from healing may perpetuate their suffering through self-destructive behaviour, such as substance abuse, or revenge and harm toward others, often leading to involvement in the criminal justice system (Walsh 2007).

Mental health issues resulting from the trauma were also common, for example, one participant specifically referred to his own suffering as ‘post-traumatic stress.’ By far, the most common issue that participants identified as coming from within themselves was uncontrollable anger. While the participants were not screened for mental health disorders as part of this study, previous research has shown mental health problems are prevalent in young inmates, with nearly one quarter (23.6 %) of incarcerated youth meeting the criteria for post-traumatic stress disorder (Dierkhising et al. 2013). Furthermore, extreme trauma during childhood increases the risk of serious problems such as oppositional disorder, depression, anxiety, risk taking and substance abuse, which in turn often leads to reactive aggression (Ford et al. 2012).

Lack of support and mental health issues often led participants to cope on their own with their psychological distress; frequently in a self-destructive manner. Evidence shows that there is a higher risk of negative behaviours such as substance abuse and destructive behaviour when painful feelings are unable to be expressed or are not supported (Walsh 2007). There are many possible reasons that these inmates did not receive treatment for mental health issues relating to these traumatic events. These may include perceived vulnerability, fear or denial of a problem; and barriers related to a man’s traditional social roles, including seeking help being unacceptable, unmanly (Tudiver and Talbot 1999). Furthermore, for many young men, a lack of knowledge or access to these services may be the reason they have not received treatment.

All of the inmates interviewed in this study reported being under the influence of alcohol and/or drugs at the time of their violent offence; and for many, there was a long history of substance abuse. Substance abuse was the principal means the participants used to mask the pain of the past, allowing them to relax and cope with, or temporarily forget their problems. As one inmate described his experience, when using heroin, it “*pulls a warm blanket over you*”. This behavior is consistent with the Self-Medication Hypothesis, whereby individuals, either being overwhelmed with pain, or being numb to their emotions, use substances to allow them to either cope or express suppressed emotions (Khantzian 1997). Invariably, for the inmates in the current study, drug and alcohol use escalated out of control and contributed directly to their violent criminal behavior.

Untreated anger or mental health issues, combined with substance use, commonly led to a ‘*brain snap*’ while under the influence of substances. Participants explained how they put up with a certain amount of provocation or frustration, but reached a point where they just exploded, and violently lashed out at their victim. Supporting this, evidence shows that offenders with highly impulsive or antisocial profiles, who were intoxicated at the time of their offence, tended to act without forethought. Instead, they impulsively snap leading to uncontrollable violent behaviour, with the immediate objective being to reduce or eliminate the seemingly overwhelming threat facing them (Declercq et al. 2012).

It is of considerable interest that the identified trajectory from childhood trauma to adult incarceration was an experience common to both Indigenous and non-Indigenous participants. Factors that have been found to predict the perpetration of violence in the general population, and that support the evidence in the current study include child/adolescent abuse; substance use in adolescence; poor quality adolescent peer networks; adversity in the family of origin, including poor relationships with parents in childhood and adolescence; witnessing parental violence; and low socio-economic status (Costa et al. 2015). Certainly, previous research has identified factors that are particularly salient for Indigenous Australians, including responses to racism and conflicting demands of communication across different cultures, forced removals and substance abuse (Homel et al. 1999; Langton 1988). While many individual, social and environmental factors influence whether an individual will commit violence and become incarcerated (Homel et al. 1999; Zubrick et al. 2010) the evidence for the trajectory reported by both cultural groups in this study is compelling.

### Limitations of this study

As only one group of inmates at one point in time were interviewed, the results of this study cannot be generalised to other similarly-incarcerated populations. However, the objective of this qualitative research was to find a small sample of very knowledgeable participants to provide an in-depth exploration of a very difficult and under-researched topic. While these participants reported a clear trajectory from trauma to incarceration, it cannot be claimed for certain that any of the events depicted were the direct causes of violence and incarceration per se, as many other possible contributing factors could have been influential in the lead up to the participants’ incarceration. However, it is noteworthy that participants, themselves, were strongly inclined to directly attribute their violent crimes and incarceration to these traumatic events and to substance abuse.

An additional limitation is that the researchers did not have full control over selection of the participants within the centre with inmates initially recruited by the residential accommodation manager. However, due to QCS and JCU ethics requirements, the recruitment method employed was the only possible procedure available for the current study. This method ensured the ethical treatment of inmates, and the health and safety of all participants, centre staff, and researchers within a challenging setting.

The recruitment procedure provided an informative group for the study with an age distribution, cultural and socio-economic background broadly similar to the prison population. Previous studies of a similar nature within juvenile detention centres in New South Wales (NSW) have been conducted (Hando et al. 1997; Kenny and Lennings 2007) but with recruitment procedures not specified. This makes it difficult to assess the study’s consistency with recruitment methods followed in similar settings.

## Conclusion

In conclusion, this paper describes the common trajectory from trauma to incarceration, as identified with male inmates at a high security correctional centre in QLD, Australia. This trajectory typically began with a specific event or prolonged experience of trauma occurring during childhood or adolescence, including but not limited to: witnessing violence; the death or absence of family members; prolonged bullying as a child; and sexual abuse. A lack of support for the trauma, along with maladaptive coping methods, commonly led to uncontrollable anger, and possible stress disorders for these inmates. Consistently, alcohol and illicit drugs were used to mask the psychological pain of the trauma. Finally, pent up frustration or anger caused many participants to eventually explode or have a ‘*brain snap*’ and commit a violent offence against another individual, ultimately leading to their incarceration.

Adding to the existing limited literature on this topic (Hando et al. 1997; Kenny and Lennings 2007); this is the first time this kind of exploratory study has been attempted with Indigenous and non-Indigenous inmates in an adult high security prison. Additionally, the current study allowed participants the opportunity to provide full and rich nuanced accounts of their experiences, an outcome that is difficult to achieve using structured surveys and scales - which has been the approach in the available literature. Although a simplified explanation of the identified trajectory is presented, it is of considerable significance that a similar path to incarceration was described by both Indigenous and non-Indigenous inmates. This implies that the high rate of incarceration for Indigenous men in QLD, and perhaps Australia wide, is not due to cultural background alone, therefore further research is required to determine what the major risk and protective factors may be. It is possible that there are higher rates of trauma experienced in Indigenous communities, contributing to the over-representation of Indigenous Australians in custody. Other unique factors, such as unresolved historical issues and family and clan rivalries (Homel et al. 1999; Langton 1988) are likely to be important and also warrant further investigation.

### Recommendations and future research

In light of these findings, early prevention and intervention should target the detection and treatment of consequences of trauma. To aid recovery from trauma, it is important to be able to come to terms with it, make sense of it, and put it into perspective using resources such as supportive family and community networks. Furthermore, early intervention is vitally important for those who have suffered trauma and traumatic loss (Walsh 2007). Leaving a traumatised person untreated may result in years of negative behaviour becoming entrenched and difficult to treat later in life. In particular, it is essential that the needs of traumatised youth be met; otherwise they will be likely to engage in self-destructive behaviour such as substance abuse, ultimately leading to violent behaviour and incarceration (Bell 2001; Dierkhising et al. 2013).

Researchers should continue to explore developmental pathways from trauma exposure to justice involvement, focusing on the timing of the trauma and cumulative exposure across developmental stages (Dierkhising et al. 2013). Additionally, an exploration of protective factors for young men faced with trauma, but not involved in the justice system is required, to discover the key differences, if any, between traumatised youth that do go on to violently offend and those who do not. As young Australian men are at particular risk of incarceration, identifying the types of trauma suffered during childhood; and devising strategies to begin to heal, warrants vigorous investigation. This may serve to minimize the devastating effects of childhood trauma, and limit the risk of many young people ending up on the predictable course to incarceration.

## Abbreviations

IPA: Interpretative Phenomenological Analysis
JCU: James Cook University
NSW: New South Wales
PTSD: Post Traumatic Stress Disorder
QCS: Queensland Corrective Services
QLD: Queensland
USA: United States of America
